# Using genomics to understand the mechanisms of virulence and drug resistance in fungal pathogens

**DOI:** 10.1042/BST20211123

**Published:** 2022-06-17

**Authors:** Miquel Àngel Schikora-Tamarit, Toni Gabaldón

**Affiliations:** 1 Barcelona Supercomputing Centre (BSC-CNS), Plaça Eusebi Güell, 1-3, 08034 Barcelona, Spain; 2 Institute for Research in Biomedicine (IRB Barcelona), The Barcelona Institute of Science and Technology, Baldiri Reixac 10, 08028 Barcelona, Spain; 3 Catalan Institution for Research and Advanced Studies (ICREA), Barcelona, Spain; 4 Centro de Investigación Biomédica En Red de Enfermedades Infecciosas, Barcelona, Spain

**Keywords:** drug resistance, genomics, infection, mycology, pathogenesis

## Abstract

Fungal pathogens pose an increasingly worrying threat to human health, food security and ecosystem diversity. To tackle fungal infections and improve current diagnostic and therapeutic tools it is necessary to understand virulence and antifungal drug resistance mechanisms in diverse species. Recent advances in genomics approaches have provided a suitable framework to understand these phenotypes, which ultimately depend on genetically encoded determinants. In this work, we review how the study of genome sequences has been key to ascertain the bases of virulence and drug resistance traits. We focus on the contribution of comparative genomics, population genomics and directed evolution studies. In addition, we discuss how different types of genomic mutations (small or structural variants) contribute to intraspecific differences in virulence or drug resistance. Finally, we review current challenges in the field and anticipate future directions to solve them. In summary, this work provides a short overview of how genomics can be used to understand virulence and drug resistance in fungal pathogens.

## Fungal pathogens, a pressing threat

Fungal pathogens (i.e. fungal organisms able to cause disease in a given host) are of high relevance for human health, affecting annually a billion people and causing 1.5 million deaths worldwide [1]. In addition, they are a threat to food crops [2] and other ecosystems, where fungal outbreaks have brought several species to the brink of extinction [3]. Fungal pathogens belong to diverse lineages of the fungal tree of life, including microsporidia, ascomycotina, basidiomycotina and mucoromycotina [4–7]. In addition, the set of species that are pathogenic to a given organism can be evolutionarily distant and have close non-pathogenic relatives, indicating that virulence has emerged multiple times independently [8,9]. Most fungal pathogens are opportunistic, meaning that they can have a commensal existence within the host or do not even require a host. This ‘plasticity’ of the pathogenic trait is also illustrated by the common emergence of new pathogenic species [10–12]. The diversity of fungal pathogens makes their identification challenging [13] and is also reflected in a variety of virulence mechanisms that are generally poorly understood. To complicate things further, antifungal drug resistance is on the rise [14–17]. As virulence mechanisms, resistance mechanisms can vary across and within species, and how resistance is acquired through evolution is poorly understood [15]. In summary, the molecular and evolutionary mechanisms behind the acquisition of virulence or drug resistance remain obscure, which constrains our ability to fight fungal pathogens.

## Genomics comes to the rescue

Phenotypes like virulence or drug resistance are ultimately dependent on genomic information. Hence, comparing genome sequences across species or isolates with different virulence or resistance profiles is a promising avenue to identify the genetic bases of these traits. Fungal pathogens have highly dynamic genomes, showing large differences across species [18–20], within species [21–24], and even within clonal populations in a host [25,26]. Such genomic changes have been linked to rapid adaptation in changing environments, likely underlying the emergence of antifungal resistance [14,27,28] and virulence [28–30].

**Figure 1.**
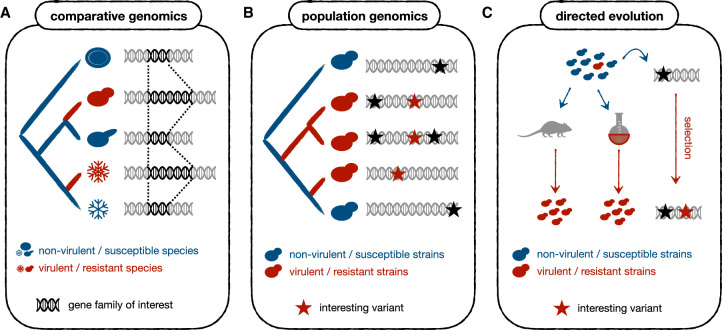
Several techniques have been used to understand the genomic drivers of drug resistance or virulence. (**A**) Comparative genomics refers to the comparison of gene content and genomic sequences across species differing in a given trait of interest (e.g. virulence towards a given host). By identifying genomic changes that correlate with the trait, hypotheses can be made on their possible relationships. This example shows a gene family with extra copies in some species, which may underlie virulence or drug resistance. (**B**) Population genomics is the comparative study of genomic variation within and across populations of a given species. This technique has been used to correlate genomic and phenotypic variation across strains of fungal pathogen species. This example shows a gene with several variants (in red) that underlie the emergence of virulence or drug resistance in some strains. Note that, since there is some divergence between strains, it is not trivial to distinguish causal (red) from passenger (black) variants. (**C**) Directed evolution experiments consist in using selective regimes (either *in vivo* (left) or *in vitro* (right)) to ‘force’ the appearance of the phenotypes under study. The selected strains are sequenced to identify variants underlying the phenotypes. This approach simplifies the detection of causal mutations as compared with population genomics studies because the evolutionary conditions are more controlled. This example represents a gene that acquired a single causal variant (in red) driving virulence or drug resistance during artificial evolution. Note that identifying causal variants here is easier (as compared with population genomics techniques (B)), because the evolutionary period is shorter and more controlled.

Compared with traditional molecular methods, current high throughput genome sequencing approaches provide a more comprehensive picture of genetic changes and do not require prior knowledge on potentially relevant loci. This has revolutionized the way in which fungal pathogens can be studied. In this review, we survey the major genomic approaches that have been instrumental in studying virulence and drug resistance in fungi, and illustrate them with specific hallmark studies. Given length limits and the large amount of relevant studies, we cannot be comprehensive. Similarly, we use a narrow definition of genomics, restricted to the study of genome sequences, and not the broader meaning including transcriptomics or epigenomics. Each of the following sections is focused on a particular genomic approach (see Figure 1).

## Comparing to learn: comparative genomics

Comparative genomics refers to the evolutionary comparison of gene content and genomic sequences across species differing in a given trait of interest (e.g. virulence towards a given host). By identifying genomic changes (e.g. acceleration, duplication, loss, or acquisition of certain genes) that correlate with the presence or the strength of the trait, hypotheses can be made on their possible relationships. Such studies are increasingly powerful given the growing availability of complete genome sequences of fungal pathogens and their close non-pathogenic relatives.

Several studies have successfully exploited comparative genomics to understand how virulence and intrinsic drug resistance have emerged in different lineages of fungal pathogens (Figure 1). For example, an analysis of Nakaseomyces species identified expansions and diversification of cell adhesion proteins as drivers of the emergence of virulence in different *Candida* species. This suggests that increased adherence favored host adaptation [12]. Similarly, the expansion of drug efflux pumps and point mutations on the azole target *ERG11* in the genome of *Candida auris* may underlie its intrinsic drug resistance as compared with closely related species [18,31]. Another example can be found in *Fusarium oxysporum*, which may have become a broad plant pathogen thanks to the horizontal acquisition of virulence genes [32]*.* In addition, some studies suggested that the pathogenic behavior of *F. oxysporum* sub-species is driven by meiotically unstable ‘accessory chromosomes’, which encode genes related to host specificity [33,34].

On another line, some studies have focused on understanding how genomic changes can drive a switch in the ecological niche of fungal pathogens, which improves our understanding about the virulence or drug resistance mechanisms. For example, a comparative study revealed that fungal pathogens had more gene-disrupting transposable element insertions than non-pathogenic species [19]. Similarly, the comparison of pathogenic and environmental *Sporothrix* species revealed that gene loss (i.e. of plant degrading enzymes and virulence inhibitors) is more important than gene gain in the evolution of pathogenesis [35]. Furthermore, it has been proposed that horizontal gene transfer of virulence genes has shaped the adaptability of *Verticillium dahliae* subspecies towards different hosts (i.e. tomato or cotton) [36]. In addition, a recent phylogenomics study proposed that the loss of mitochondrial complex I in some fungi (i.e. *Candida glabrata* [37]) may have generated increased tolerance to oxidative stress, predisposing them towards intrinsic drug resistance [38,39].

## Zooming in: population genomics

Population genomics refers to the comparative study of genomic variation within and across populations of a given species. Once a reference genome of a species is available, population genomics can be performed by sequencing additional individuals to identify genetic variants. The frequency and distribution of such variants can inform about the genetic structure of a species, identify sub-clades, reconstruct the history of populations, and identify genomic regions under selection. Fungal pathogens can display large genetic and phenotypic variation across different strains of the same organism [21–23,25,26]. Population genomics techniques have been used to correlate genomic and phenotypic variation across isolates of fungal pathogen species (Figure 1).

Most population genomics studies reconstruct a phylogeny showing the relationships between the isolates, which helps to understand the evolutionary process underlying the emergence of a given phenotype of interest. The populations of most fungal pathogens include clearly separated clades, and some of these clades have particular drug resistance or virulence properties [21,23,40]. For example, some clades of *Cryptococcus neoformans* are particularly virulent, possibly through resistance to oxidative stress [21]. Similarly, there are clades in *Candida auris* and *Candida albicans* that lost the ancestral azole drug resistance [22] and virulence capabilities [40], respectively. Conversely, there is not a clear association between clade identity and antifungal resistance patterns in *C. glabrata* [23,41], or *Aspergillus fumigatus* [42].

Beyond describing when and where phenotypes appeared, population genomics techniques have been used to infer underlying mechanisms. The most simple approach involves testing specific hypotheses about mutations contributing to a given phenotype, which are formulated from known mechanisms of drug resistance or virulence. For example, a recent study in *Candida auris* found that *ERG11* (the target of azoles) mutations and copy-number variants are associated with azole resistance, while *FKS1* (echinocandin target) mutations are related to echinocandin resistance [22]. Another study in *Candida glabrata* found that *FKS1* mutations are related to echinocandin resistance, while *PDR1* and *CDR1* mutations are associated with azole resistance [41].

However, such approaches mostly investigate a few genes, and Genome-Wide Association Studies (GWAS) are a promising, more comprehensive, alternative. GWAS was used to find variants associated with azole resistance in *Candida glabrata* (two regulatory SNPs in *CST6* [43]) and *Aspergillus fumigatus* (two missense mutations in a sterol-modulator gene [42] and missense mutations in ABC transporters and mitochondrial proteins [44]). In addition, GWAS comparing environmental and clinical isolates of *Cryptococcus neoformans* revealed major differences in virulence and oxidative stress-related genes, potentially involved in pathogenesis [21]. Similarly, a GWAS study on *Aspergillus fumigatus* isolates found that variants in genes related to growth in hypoxia, iron homeostasis and regulation of gluconeogenesis are linked to clinical phenotypes [45]. Finally, GWAS comparing virulent and avirulent strains of the wheat pathogen *Zymoseptoria tritici* revealed tens of small variants and rearrangements underlying pathogenesis [46]. Importantly, some of these processes affect isolates of human pathogens from the same patient [25,26], suggesting that real-time monitoring could be key for an optimal treatment.

## Seeing it live: directed evolution

While population genomics can be powerful, it faces two main limitations for understanding the mechanisms of drug resistance or virulence. First, the study of natural variation is mostly useful for phenotypes that emerged independently several times in a population. For example, population genomics may be underpowered to study drug resistance of a recently approved compound (i.e. beauvericin [47]), the virulence mechanisms of an emergent fungal pathogen (i.e. *Candida blankii* [48]) or the genomic drivers of a phenotype that emerged only once in the population (i.e. the virulence loss at the common ancestor of the isolates in clade 13 from *Candida albicans* [40]). Second, large divergence between isolates with different phenotypes complicates the (key) distinction between causal and passenger mutations (as reviewed in [49]).

Directed (artificial) evolution (either *in vitro* [50,51] or *in vivo* [52]) of drug resistance or virulence followed by whole-genome sequencing (WGS) of the adapted strains offer a promising solution to overcome some of these problems. In directed evolution experiments the conditions are controlled, and the phenotypes under study are ‘forced' to appear in otherwise wild type strains by using selective regimes. This means that phenotype-causing mutations are expected to be fixed in the selected populations and, if the process is repeated, they are expected to appear recurrently. This usually simplifies the detection of such mutations as compared with population genomics studies (Figure 1). Such approaches have been used to understand the *in vitro* evolution of azole and echinocandin resistance in *Candida glabrata*, which revealed genes related to resistance and cross-resistance [53]. In addition, similar experiments were performed in *Candida auris* [54,55], *Candida parapsilosis* [56] and *Candida albicans* [57].

The mechanisms of virulence have also been partially studied through directed *in vivo* evolution experiments. A study of *Candida albicans* evolved avirulent strains (starting from a virulent parental) in murine models and found that changes in *EFG1* and *FLO8*, related to hyphal growth, were related to the loss of virulence [52]. This exemplifies how evolution in the host can yield strains with lower virulence [58]. Another study found that passing *C. albicans* through murine models (*in vivo*) results in highly diverse populations, which revealed genomic changes underlying host adaptation [59].

A similar approach is the use of random mutagenesis coupled to sequencing of mutants with a particular phenotype to ascertain the underlying genomic changes. For example, chemical mutagenesis (with ethyl methanesulfonate) followed by whole-genome sequencing was used in *Puccinia* species to understand their virulence mechanisms towards wheat [60,61]. In addition, genome-wide transposon-mediated mutagenesis revealed that the lncRNA *DINOR* is a regulator of stress responses (including drug tolerance) in *Candida auris* [62].

In summary, using directed evolution or random mutagenesis coupled with genome sequencing has allowed the exploration of drug resistance and virulence in controlled settings. However, such methods may not recapitulate entirely the natural evolutionary process. For example, natural evolution of drug resistance or virulence involves synergistic action of multiple selective forces that may be absent from some artificial settings settings. We thus consider that complementary approaches (such as population genomics) are also key to obtain the complete picture. The following sections describe some of the genetic alterations linked to these phenotypes through directed evolution experiments and population genomics techniques.

## Small variants bringing large change

**Figure 2.**
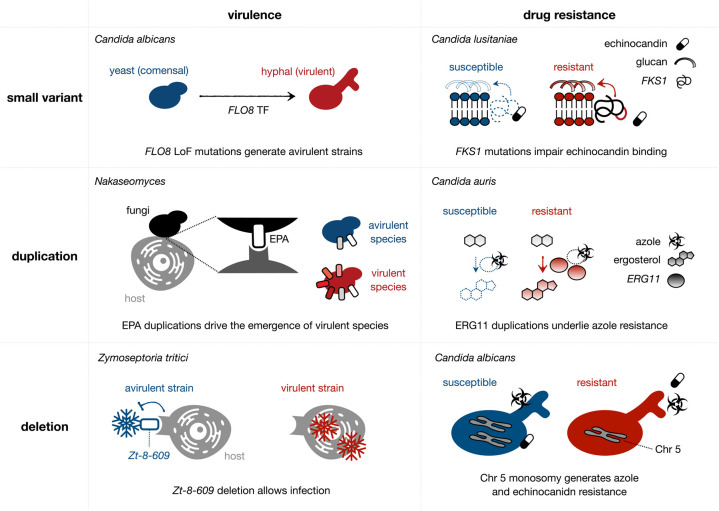
Several types of genomic changes can drive the acquisition of virulence or drug resistance. We show illustrative examples of how small variants (top), duplications (middle) and deletions (bottom) can alter virulence (left) or drug resistance (right) across species or within isolates of the same species. This figure is a graphical support to the sections ‘Small variants bringing large change’ and ‘Do not neglect structural variants’ of the main text.

Most research linking genomic variants to drug resistance or virulence is focused on finding Single Nucleotide Polymorphisms (SNPs) and small insertions/deletions (INDELs) between isolates of a given fungal pathogen species. These strains may be different isolates from a population [21–23], pairs of parent-daughter lineages from a directed evolution experiment [52,54] or serial isolates from a given patient [25,26]. To identify such ‘small’ variants, most studies use custom pipelines that include mapping of sequencing reads to a reference genome and a variant calling step, which is performed with algorithms like GATK [63] or freebayes [64]. This is usually followed by variant annotation (using tools like VEP [65] or SNPeff [66]) to prioritize candidate mutations. Some automatic pipelines that call and interpret small variants directly from the reads have been developed to simplify this task, such as YMAP [67] and perSVade [68], which have been specifically developed for fungi. Below we describe some examples of how small variants can generate drug resistance and virulence mechanisms (see Figure 2).

Missense mutations in the target enzymes may impair the drug binding and cause resistance. For example, echinocandin resistance is caused by missense changes or codon deletions in the *FKS* genes of *Candida glabrata* [53,69], *Candida auris* [22,55], *Candida lusitaniae* [70] and *Candida albicans* [71]. Similarly, mutations in *ERG11* gene (involved in ergosterol biosynthesis) have been associated with azole resistance in *Candida glabrata* [53], *Candida albicans* [71] and *Candida auris* [22]. In addition, small variants affecting other proteins of the ergosterol biosynthesis have been linked to multidrug resistance through unknown mechanisms. Loss-of-Function (LoF) variants in *ERG3* have been linked to both azole and echinocandin resistance in *Candida glabrata* [53], *Candida parapsilosis* [56,72], and *Candida albicans* [71], and polyene resistance in *Candida lusitaniae* [70]. In addition, missense and regulatory SNPs in ergosterol biosynthesis genes (i.e. *atg26*) have been linked to azole resistance in *Aspergillus fumigatus* [42].

Another resistance mechanism involves the hyperactivation of drug tolerance pathways through Gain-of-Function (GoF) mutations. For example, azole resistance in *Candida glabrata* and *Candida auris* are often caused by GoF mutations in *PDR1* (*C. glabrata*) or *TAC1b* (*C. auris*) transcription factors (TF), driving the overexpression of the *CDR1* drug efflux pump [26,53–55,71,73]. Similarly, (potentially GoF) *CDR1* missense mutations have also been linked to azole resistance [53,71].

Small variants have also been linked to changes in virulence. For example, GoF changes in *PDR1* drive *EPA1* (adhesin) overexpression and higher host adherence [26]. This mutation also caused azole resistance, which exemplifies how a single variant can drive both phenotypes. Similarly, LoF mutations in the TF *BZP4* of *Cryptococcus neoformans* reduced melanization and virulence [21]. In addition, LoF small variants in the TF *FLO8* were associated with reduced hyphal growth in *Candida albicans* avirulent strains [52]. Although the recurrent presence of mutations associated with a phenotype is a strong mechanistic hint, the causative relationship can be experimentally validated through subsequent re-introduction of the mutations in a naive background or replacement of the wild type alleles in the mutant. This underscores the power of combining genomic and molecular biology techniques.

## Do not neglect structural variants

Beyond small variants, complex Structural Variants (SVs) (i.e. chromosomal aneuploidies, duplications, deletions, inversions and other rearrangements) have been shown to modulate differences in drug resistance and virulence across strains of fungal pathogens. Most current studies analyzed the role of a subset of SVs, copy-number variants (CNVs), identified from changes in genomic read depth.

On the one hand, whole-chromosome aneuploidies (losses or gains) have been linked to azole resistance in *Candida glabrata* [53], *Candida auris* [54] and *Candida albicans* [74], likely due to overexpression of genes encoding drug target enzymes (which lower the impact of the drug) and/or efflux pumps (reducing the intracellular drug concentration). Interestingly, such aneuploidies may generate multidrug resistance for compounds with different mechanisms of action. For example, a study in *Candida albicans* found that chromosome 2 trisomy promotes resistance to hydroxyurea and caspofungin, while chromosome 5 monosomy generates resistance to azoles and echinocandins [74]. Similarly, most aneuploidies in *Candida albicans* yielded condition-specific fitness benefits [75], suggesting they are major drivers of adaptive evolution. This has also implications for the emergence of virulence. For example, it has been proposed that aneuploidies in the forest pathogen *Dothistroma septosporum* drive increased levels of dothistromin (a mycotoxin), resulting in strains with higher virulence [76]. In addition, plant-associated (virulent) isolates of *Phytophthora ramorum* (oak pathogen) had more aneuploidies than *in vitro*-grown strains, suggesting that they underlie virulent phenotypes [77].

On the other hand, smaller CNVs (i.e. duplications generating overexpression of *ERG11* or *CDR1* genes) have been linked to azole resistance in *Candida glabrata* [73], *Cryptococcus neoformans* [21], *Candida albicans* [78] and *Candida auris* [22]. Such CNVs have also been linked to changes in virulence, including CNVs in adhesins [43] or secreted aspartyl proteases [79] in *Candida glabrata*, or CNVs in cell wall and stress-response genes in *Candida auris* [80]. Another example was found in the plant pathogen *Zymoseptoria tritici*, where virulence was associated with a small deletion breaking the Zt_8_609 gene, potentially involved in a specific interaction with a plant resistance gene. This interaction protects the plant from infection, so that the deletion is key for virulence [46]. This further illustrates how gene loss can drive important phenotypes in fungi.

In summary, read-depth based CNV calling revealed that SVs play a fundamental role in fungal pathogens. However, the technique itself has several limitations. First, such CNV calling has limited resolution (often ignoring small CNVs) and lacks precision in defining breakpoints positions [53,54]. Second, read depth can be noisy and biased by factors like GC content, read mapping errors [67,81] or the distance to the telomeres [67], which limit accuracy. Third, CNVs are only a subset of all SVs, which means the role of more complex SVs (like inversions or translocations) has been overlooked. This is likely due to difficult SV detection from short reads, which may be solved by using long reads [82] or recent methods for accurate short read-based SV calling [68,83,84]. For example, a recent study in *Candida glabrata* based on short reads found that translocations around *FKS* and *ERG11* genes may play a role in echinocandin and azole resistance, respectively [53]. These results indicate that further research should consider the role of complex SVs in drug resistance and virulence of fungal pathogens. Figure 2 provides a graphical representation of how SVs generate such phenotypes.

## Challenges and future directions

Genomic techniques face several limitations, and we enumerate here the most important ones, together with future directions that may solve them.

On one hand, the functional interpretation of genomic changes relies on annotations which are often suboptimal, mostly based on homology-based predictions. Further experimental work and better annotation tools may solve this. Similarly, the impact of missense mutations on protein function is difficult to predict, which hampers such functional interpretation. This could be addressed with variant annotators that rely on structure information (i.e. FoldX [85]) based on sequence-based structure predictions (i.e. AlphaFold2 [86]). In addition, using CRISPR-Cas9 can be key (as done in [87] or [53]), since it allows to reproduce mutations in isolation to study their phenotypic impact, even in non-model species.

On another hand, the typical approaches could be missing important genomic variation. Many studies may be underpowered because of their small sample sizes. Combined analyses of genomic and phenotypic information from different studies may increase this power and reveal novel mechanisms of drug resistance and virulence. In addition, the contribution of SVs to these phenotypes has been often ignored, likely due to the technical difficulties in calling them from short-read data. This could be solved by using recent short read-based SV callers (i.e. perSVade [68]) or long read sequencing. Similarly, the epistatic interactions shaping the genotype-to-phenotype relations are poorly understood, although they could be very important [88]. Increasing sample sizes and considering strains of genetically distinct clades may be essential to fully characterize how genomic changes drive virulence and drug resistance. Finally, most GWAS studies only evaluated the correlation between specific variants and a given phenotype. This approach can be underpowered to detect genes related to resistance or virulence, since different variants in the same gene may have similar phenotypic effects [53]. The use of collapsing GWAS methods [89], which group variants into functional categories (i.e. genes or pathways) to test groups instead of variants, may be a useful solution.

Another setback is that the generated knowledge does not always have a direct clinical impact for human pathogens. Further efforts are required to apply this knowledge and approaches to monitor the phenotypes in the clinics or design better treatment guidelines. We consider that addressing these limitations will be necessary to understand these phenotypes and fight fungal pathogens.

## Perspectives

- Fungal pathogens pose a growing threat to human health, food security and ecosystem diversity. We have shown how genomic studies have been instrumental to understand the mechanisms of virulence and drug resistance in order to fight these pathogens.
- Comparative and population genomics studies can reveal associations between naturally occurring phenotypes and underlying mutations. However, their power can be limited if the compared strains are too divergent, and directed evolution coupled with genome sequencing can overcome this limitation.
- These approaches have revealed that small variants, mostly affecting protein sequences, often underlie drug resistance and virulence phenotypes. In addition, structural variants can also contribute to these phenotypes, although their role is still poorly understood.

## Abbreviations

CNVs: copy-number variants
GWAS: genome-wide association studies
SNPs: single nucleotide polymorphisms
SVs: structural variants
TF: transcription factors
